# Innovative Chemical Process for Recycling Thermosets Cured with Recyclamines^®^ by Converting Bio-Epoxy Composites in Reusable Thermoplastic—An LCA Study

**DOI:** 10.3390/ma11030353

**Published:** 2018-02-28

**Authors:** Angela D. La Rosa, Ignazio Blanco, Diosdado R. Banatao, Stefan J. Pastine, Anna Björklund, Gianluca Cicala

**Affiliations:** 1 Department of Civil Engineering and Architecture and INSTM UdR, University of Catania, Viale A. Doria 6, 95125 Catania, Italy; gcicala@unict.it; 2 Connora Technologies Inc., 30621 San Antonio St, Hayward, CA 94544, USA; desi@entropyresins.com (D.R.B.); stefan@connoratech.com (S.J.P.); 3 Environmental Strategies Research-fms, Royal Institute of Technology, KTH, 100-44 Stockholm, Sweden; anna.bjorklund@abe.kth.se

**Keywords:** polymer recycling, LCA, epoxy recovery, Vacuum Resin Transfer Moulding

## Abstract

An innovative recycling process for thermoset polymer composites developed by Connora Technologies (Hayward, CA, USA) was studied. The process efficacy has already been tested, and it is currently working at the plant level. The main aspect investigated in the present paper was the environmental impact by means of the Life Cycle Assessment (LCA) method. Because of the need to recycle and recover materials at their end of life, the Connora process creates a great innovation in the market of epoxy composites, as they are notoriously not recyclable. Connora Technologies developed a relatively gentle chemical recycling process that induces the conversion of thermosets into thermoplastics. The LCA demonstrated that low environmental burdens are associated with the process itself and, furthermore, impacts are avoided due to the recovery of the epoxy-composite constituents (fibres and matrix). A carbon fibre (CF) epoxy-composite panel was produced through Vacuum Resin Transfer Moulding (VRTM) and afterwards treated using the Connora recycling process. The LCA results of both the production and the recycling phases are reported.

## 1. Introduction

Recently, several techniques have been developed in order to induce the depolymerisation of thermosetting resins contained in composite materials to make them recyclable [1,2]. After depolymerisation, the resins dissolve and inorganic substances such as metal, glass fibre (GF), and CF can be separated and recovered [3,4,5,6,7,8,9].

In this field, a series of recyclable amine-based epoxy curing agents, called Recyclamines^®^, have been developed by Connora Technologies in the last few years. The generic design of these recyclable epoxy molecules is derived from a generic polyamine structure as with conventional amine epoxy hardeners. The innovation is that in Recyclamines^®^ the amino end groups are tethered together by a central cleavable group. The central programmed cleavage group is key to recyclability by converting the epoxy resin into epoxy thermoplastic. The conditions required to quickly induce crosslink cleavage in the epoxy are a combination of temperature (70–100 °C) and pH (acidic).

The Connora chemical recycling process is low energy (70–100 °C) and low cost compared to other on-going chemical or thermal processes. For example, one chemical process was developed by Hitachi [10] using benzyl alcohol as solvent and tricalcium phosphate (Ca_3_PO_4_) as catalyst. The temperature required to dissolve the composites fiber reinforced polymers (CFRPs) was 200 °C for about 10 h.

Toray Industries, Inc. (Tokyo, Japan), Teijin Ltd. (Tokyo, Japan) and Mitsubishi Rayon Co., Ltd. (Tokyo, Japan) are studying recycling technologies using a thermal decomposition technique in which resins are decomposed and removed at 500 to 700 °C [11,12].

Okajima, et al. of Shizuoka University are carrying out a research project sponsored by the New Energy and Industrial Technology Development Organization (NEDO) to study CFRP recycling technologies using supercritical alcohol [13,14].

Goto, et al. of Kumamoto University are studying a method of recycling CFRP using subcritical alcohol. When high-boiling alcohol such as benzyl alcohol is heated at 300 to 400 °C to turn it subcritical, and used for CFRP treatment, the whole resin decomposes within an hour [15,16].

None of the recycling technologies has yet come into commercial use.

## 2. Description of the Recycling Process

**Figure 1:**
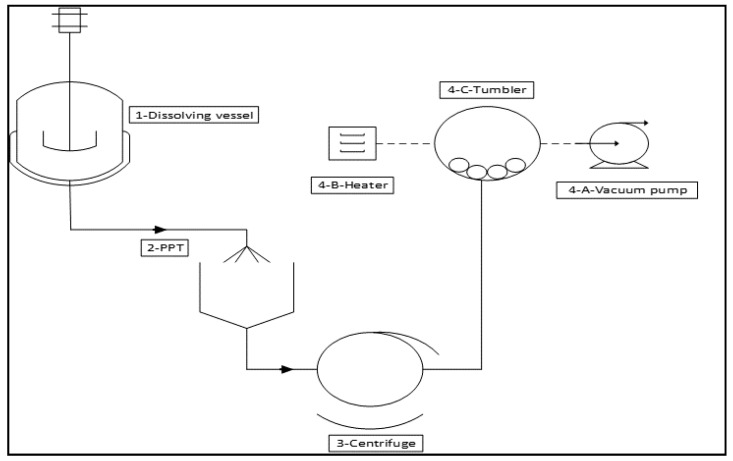
Scheme of the Connora chemical recycling process at plant scale.

The Connora recycling process performs a low-energy, solution-based recycling method for Recyclamine^®^ composites that are able to reclaim both the thermoplastic and the carbon fibers. Cured epoxy composites were placed in a dilute (25%) acetic acid recycling bath of 70 °C for 1 h. Once the thermoset matrix is fully dissolved, carbon fibres can be removed from the recycling solution, rinsed in water, and dried at room temperature. To reclaim the epoxy thermoplastic, the acidic recycling solution is neutralized with sodium hydroxide base (NaOH). The thermoplastic, which is insoluble in water, then precipitate out of solution, is filtered, rinsed, and then dried into a powder. Physical properties of the reclaimed thermoplastic were measured. The use of dilute acetic acid as a recycling solution is chemically less harsh while still being specific to recycling. The conversion of the programmed epoxy into its thermoplastic counterpart can range from 30 min to several hours depending on the composite thickness. As the thermoset matrix is cleaved and turned into the resulting thermoplastic, it remains completely soluble in the acidic recycling solution. This is due to protonation of the resulting thermoplastic polymer backbone. Thus, any other articles in contact with the initial thermoset (fibres, metal, etc.) may be easily and cleanly removed from the recycling bath. The reclaimed fibres and materials are then washed and dried for reuse. Finally, the thermoplastic is easily reclaimed as a precipitate by neutralizing the recycling solution; this phase is indicated in the present paper as PPT (thermoplastic precipitation). At, this point the recycling solution is essentially salt water and does not represent an additional waste stream. A scheme of the process is reported in Figure 1. Materials used and electricity consumption are summarised in Table 1 and Table 2.

### 2.1. Process Details and Assumptions

#### 2.1.1. Dissolving Phase

- Heating the solution to 70 °C
  - Initial temperature of the acetic acid solution and composites is 25 °C.
  - Heat capacity of water: 4.2 KJ/Kg °C.
  - Heat capacity of Acetic acid: 2.0 KJ/Kg °C.
  - Heat capacity of composite: 1 KJ/Kg °C.
  - Energy to heat the vessel to 70 °C plus the wasted energy assumed to be 10% of required energy to heat the solution.
- Keeping the solution temperature at 70 °C

The required energy to keep the system at 70 °C for each one hour is assumed 5% of required energy to heat the system from 25 to 70 °C.

#### 2.1.2. Thermoplastic Precipitation Phase (PPT)

The energy in this step is negligible.

#### 2.1.3. Washing Phase

The centrifuge power is 3 HP. For each 18 kg of epoxy, one hour of centrifuging is required.

#### 2.1.4. Drying Phase

Electricity consumption:

- Vacuum: 1 HP
- Tumbler: 1.5 HP
- Heater: 800 W

For each one kg of composite 1 h of drying process is required.

### 2.2. Reclaimed Materials

#### 2.2.1. Carbon Fibres

Due to the mildness of the recycling conditions, we can expect that the recovered carbon fibres will keep their high quality without losing their physical properties. We are currently testing this hypothesis by evaluating the properties of both chemically recycled and virgin carbon fibres in cured composites.

#### 2.2.2. Epoxy Thermoplastic Properties

Epoxy-thermoplastics possess excellent mechanical and adherent properties, and share some similar attributes with cross-linked epoxy [17,18]. However, epoxy-thermoplastics can be melt processed and are amenable to standard thermoplastics processing techniques. We are currently evaluating the ability to fabricate thermoplastic composites using the reclaimed Recyclamine-based epoxy thermoplastic. Representative properties of the reclaimed epoxy thermoplastic from recyclable epoxy are provided in Table 3. These properties are referred to as an average epoxy thermoplastic obtained after treating different end of life products, made of composites that contain epoxy resins cured by recyclamines. These composites can present a full range of properties depending on the specific formulation used. We studied, for example, two different formulations for High Pressure Resin Transfer Molding (HP-RTM) and Vacuum Resin Transfer Molding (VARTM), which displayed *T*_g_ of 56 °C and 100 °C, respectively [19,20]. The differences of *T*_g_ are due to the use of slightly different formulation to accomplish the different cure profile.

## 3. LCA Method Description

The LCA methodology is considered the most widespread technique for evaluating the environmental impacts associated with material products that analyzes the impact of any product over a lifetime from the extraction of raw materials to the waste disposal of its various components [21]. An LCA includes the following main important steps: materials extraction, manufacturing and waste production, packaging, transportation, product use, and product disposal. A life cycle inventory (LCI) is compiled to record the emissions and resources consumed that can be attributed to a specific product. For the present study, the software SimaPro 8.01 (PRé Sustainability, Amersfoort, The Netherlands) [22] was used, as well as the Ecoinvent v3 database [23]. LCA is governed by specific standards from the International Organization for Standards (ISO). The life cycle assessment study consists of defining the scope and goal, inventory analysis, assessing the impact, and interpreting the data. ISO 14040 (SO 14040, 2006) and ISO 14044 (ISO 14044, 2006) Environmental Management Life Cycle Assessment provide the principles, framework, requirements, and guidelines to conduct a life cycle assessment study [24,25].

### 3.1. Goal and Scope and Inventory Analysis

Goal and scope of the study is the evaluation of the impacts related to the chemical recycling process developed by Connora Technologies. A working batch used to chemically treat 35 kg of product was considered as functional unit. The inventory data of the chemical recycling process at plant level are listed in Table 4.

The system boundary of the present study also includes the production phase of the CF-composite panel that is chemically treated for recycling. The production process and the chemical physical characterization of this panel were already reported in previous papers [19,20]. Here, we present the environmental impacts associated with the production phase and the recycling phase of the panel, by means of the LCA method. The inventory data or the CF-panel production is reported in Table 5. Primary data were taken at plant level for the recycling process and at laboratory level for the panel production. The Ecoinvent database was used for materials and energy consumption. For the inventory of CF, the starting material polyacrylonitrile (PAN) fibres were taken from the Ecoinvent database. The electricity consumption to obtain CF from PAN was taken from literature. The epoxy-thermoplastic is not included in the Ecoinvent database; therefore, an assumption was made: it was assimilated to polycarbonate, as it displays similar properties to thermoplastic materials. A previous LCA study based on the Connora process at laboratory scale was already published [26].

### 3.2. Life Cycle Impact Assessment (LCIA)

The impact assessment involves using the inventory analysis results to come to relevant midpoint and endpoint indicators. Midpoint indicators might include acidification, radiation, climate, fossil fuel, ecotoxicity, etc. Endpoint indicators can include respiratory diseases, seawater level, resource depletion, etc.

The environmental issues analysed in a Life Cycle Impact Assessment (LCIA) are called environmental impact classification factors, as outlined in ISO/TR 14047 (2003). A brief description is provided.

Acidification Potential (AP) is a consequence of acids being emitted to the atmosphere and subsequently deposited in surface soils and waters. AP classification factors are mainly based on the contributions of sulphur dioxide (SO_2_), nitrogen oxides (NO_x_), hydrochloric acid (HCl), ammonia (NH_3_), and hydrofluoric acid (HF), and are expressed as SO_2_ equivalent.

Aquatic Toxicity Potential (ATP) is calculated based on the maximum tolerable concentrations of different toxic substances in water by aquatic organisms.

Human Toxicity Potential (HTP) is calculated in kg and takes into account the releases of materials toxic to humans in air (A), water (W), and soil (S) based on the human toxicological factors.

Eutrophication Potential (EP). Eutrophication is referred to as the pollution state of aquatic ecosystems in which the over-fertilisation of water and soil has turned into an increased growth of biomass. EP is calculated in kg based on a weighted sum of the emission of nitrogen and phosphorus derivatives such as N_2_, NO_x_, NH_4_^+^, PO_4_^3−^, P, and chemical oxygen demand (COD). The classification factors for EP are expressed as phosphates equivalents.

Global Warming Potential (GWP) is calculated for each different greenhouse gas (GHGs) (CO_2_, N_2_O, CH_4_) and volatile organic compounds (VOCs). GWP is expressed as CO_2_ equivalent.

Non-Renewable/Abiotic Resource Depletion Potential (NRADP) is calculated for fossil fuels, metals, and minerals by dividing the quantity of resources used by the estimated total world reserves of that resource.

Ozone Depletion Potential (ODP) represents the potential of depletion of the ozone layer due to the emissions of chlorofluorocarbon (CFC) compounds and other halogenated hydrocarbons.

Photochemical Oxidants Creation Potential (POCP): Photochemical smog is caused by the degradation of VOCs and nitrogen and is expressed in kg of ethylene.

The output of the LCIA consists of a list of such category indicators; it does not provide an evaluation of actual environmental impact. All emissions are sorted into classes according to the effect they have on the environment. For example, chemicals that contribute to the greenhouse effect or to ozone layer depletion are divided between those two classes, whereas emissions such as nitrogen oxides may simultaneously belong to several classes, such as aquatic toxicity, acid rain, and eutrophication. This is called Classification. The following step is called Characterisation; it means to assign and convert LCI results into numerical indicator results.

## 4. LCA Results and Discussion

### 4.1. Results of the Recycling Process

**Figure 2:**
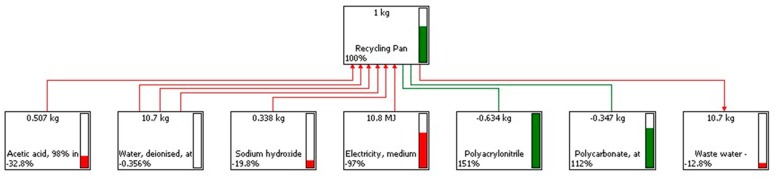
Network of the Connora recycling process according to the inventory data of Table 4. Percentage of GWPs is reported for each step according to the CML 2000 method.

Several LCIA methods were used for the impact interpretation. Figure 2 reports the network created with CML 2000, which is associated with the recycling Connora process. The output refers to the materials and electricity required to treat 1 kg of product by using this process. The red lines indicate created impacts, as they are associated with the use of chemicals and electricity. The green lines indicate avoided impacts, as they are associated with the recovered materials (PAN and the epoxy-thermoplastic). From a quick look at this network, it is understandable that the main impact associated with the electricity consumption to run the process is totally compensated for by the avoided impact due to the recovered materials.

**Figure 3:**
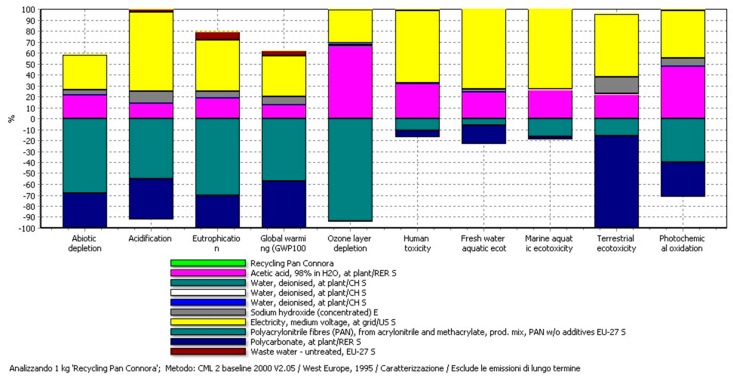
Network of the Connora recycling process according to the inventory data of Table 4. Percentage of GWPs are reported for each step according to the CML 2000 method.

A wider view of the impacts is reported in Figure 3. In this case, all the impact categories are shown according to the CML 2000 impact assessment method. For each impact category there is a strong contribution associated with the recovery of PAN and the thermoplastic, with values below zero. These avoided impacts in all cases are able to minimize the positive impacts created mainly by the use of acetic acid and electricity consumption. For example, looking at the GWP and the Eutrophication columns, the total impact results are negative, meaning that the avoided part is bigger than the created one.

### 4.2. Results of the CF-Panel Production

As already mentioned in this paper, the great innovation and utility of the Connora process is the possibility of dissolving epoxy resins and recycle thermoset composites. Polymeric matrices are divided into thermoplastics and thermosets, thermoplastics can be melted by heating and solidified by cooling, whereas thermosets, once polymerized, cannot be re-melted or re-formed being thus not suitable for recycling. These polymeric matrices were mainly landfilled or incinerated, contributing to an increase in the environmental impact at the end-of-life. As the market for the epoxy composites is still growing, since they are the preferred choice for aircraft and car structures [27], it is very important to develop and optimize recycling processes at industrial level. In order to evaluate the impact of the Connora process as end-of life method for CF-composites, we carried out an environmental evaluation of the production phase of a CF-panel made with Recyclamie-epoxy system.

**Figure 4:**
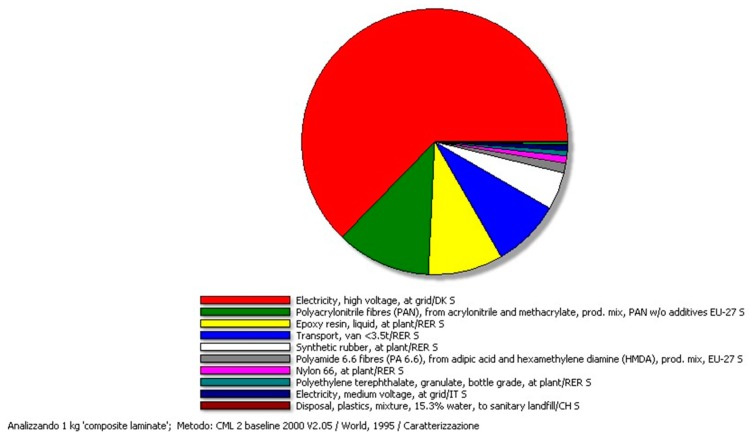
Production of CO_2_ associated with the CF-composite. Electricity consumption, PAN, and epoxy resin are the main contributors.

Diagram in Figure 4 clearly reports the main contributions to the impact for the production of the CF-composite panel, which are the electricity consumption the highest amount followed by materials use (PAN and the epoxy resin) and materials transportation.

**Figure 5:**
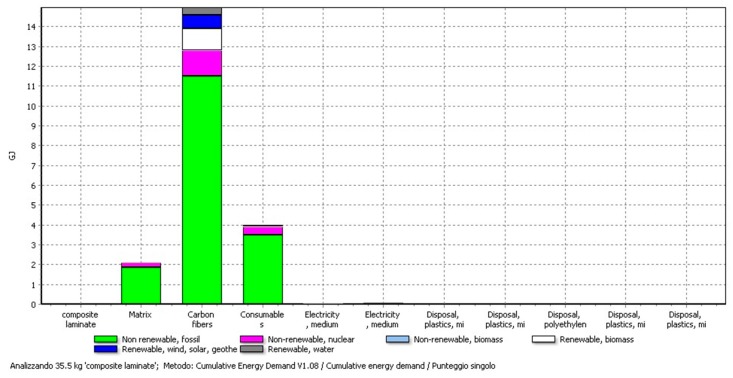
Electricity consumption in the CF-composite preparation. The highest contribution comes from the production of carbon fibres.

More specifically, in Figure 5 it is possible to understand that the electricity consumption derives mainly from the carbon fibres; in fact, the CED value is almost 15 GJ for FC and between 2 and 4 GJ for the other materials (epoxy matrix and consumables used for the process).

**Figure 6:**
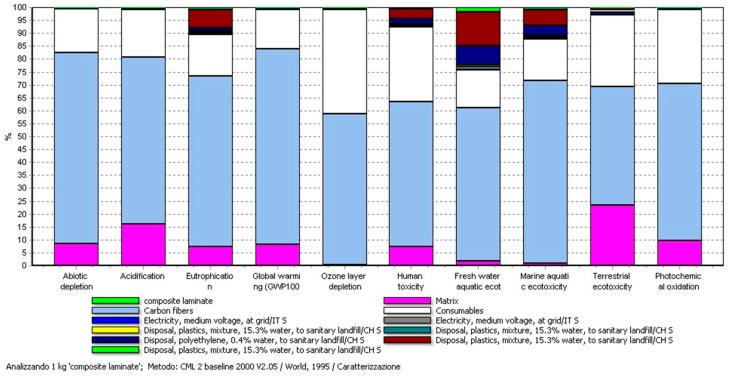
Impacts associated with the production of the CF composite laminate, method CML2000. For all the impact categories, the main contributions derive from the use of CFs, the matrix (epoxy resin + Recyclamine^®^), and the consumables required for the VRTM process.

The same trend is extended to all the other impact categories as shown in Figure 6.

### 4.3. LCA Results of the Production and the Recycling Phases

**Figure 7:**
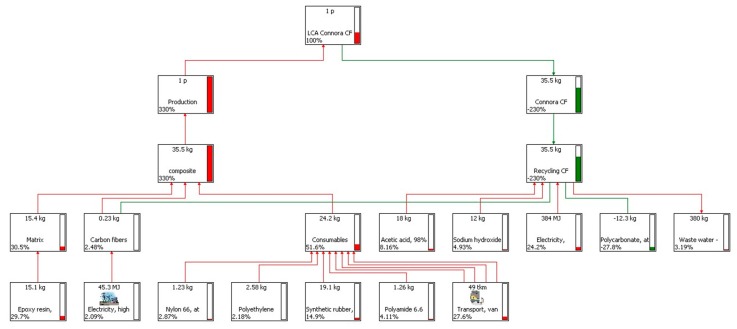
LCA diagram of the production and recycling of the CF-laminate. Scenario (a): the CFs are recovered and reused as they are.

**Figure 8:**
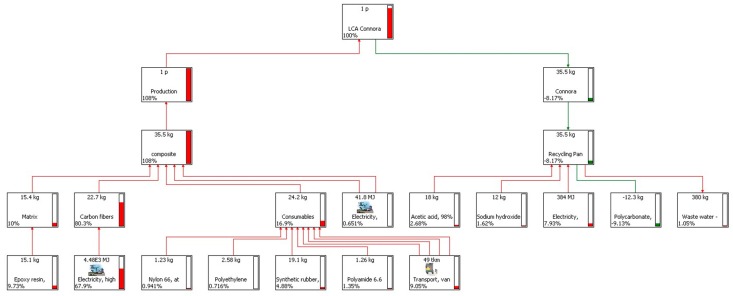
LCA diagram of the production and recycling of the CF-laminate. Scenario (b): the CFs are recovered as PAN and need further treatment to be re-used as CFs.

LCA networks of CF-panel production and end-of-life are shown in Figure 7 and Figure 8. As we consider a key factor for the CF-recycling process the quality of the recovered CFs, we assumed two scenarios.

Scenario (a): In this case, the CFs recovered after the Connora process are reused without need of further treatment to improve their mechanical properties. We assume that the recycling process will not affect the properties of the CFs that are used to make the panel. In this case, the amount of avoided impact is incredibly high.

Scenario (b): In this case, the CFs are recovered but they result more poorly in terms of their chemical-physical and mechanical properties. They require further processing, with the consumption of electricity, in order to be reused to replace virgin CFs. In this scenario, we assume that the quality of the recovered CFs is at the level of PAN. The avoided impacts in this case are due to the avoided use of virgin PAN as raw material in the production of new CF composites.

**Figure 9:**
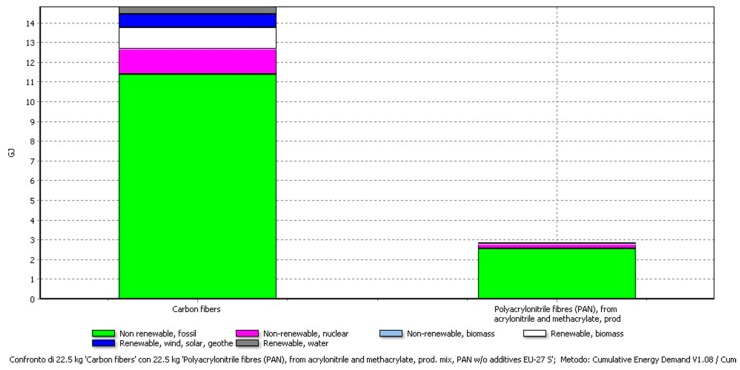
Cumulative energy demand that is required to produce Carbon fibres and PAN. Data for PAN were taken from the Ecoinvent database included in SimaPro. For CFs, the energy consumption was taken from literature.

A comparison of the different amounts of electricity required to produce PAN and CFs is reported in Figure 9 and Table 6. The CED values refer to the amount of 22.49 kg that is the amount of recovered CFs from the Connora process. The big difference in the values is due to the process required to obtain the CFs from PAN.

This gives a measure of the avoided impact when recovering the CFs, assuming scenarios (a) and (b). A comparison of all avoided impacts considering both scenarios is reported in Table 7.

An avoided impact of kg CO_2_ eq (−779) is obtained when recycling CFs “ready to use”, while the avoided impact is −84.5 kg CO_2_ eq when the CFs are recovered at the level of PAN.

**Figure 10:**
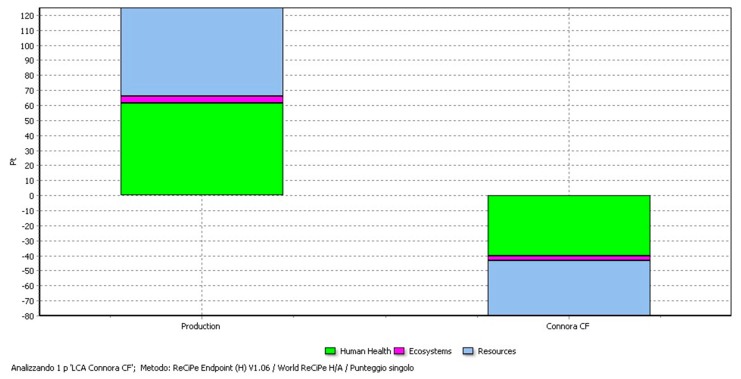
Damage assessment through ReCiPe Endpoint. Avoided damages on Human Health (HH), Ecosystems, and Resources are associated with the recycling process with recovery of CFs.

An evaluation of the damage assessment is reported in Figure 10 using the ReCiPe Endpoint method. The damage created during the production phase is to a large extent balanced by the materials recovery during the recycling process, especially when the CFs are recovered at a “ready to use” level.

## 5. Conclusions

For many years, composite waste has primarily been disposed of in landfills but now the EU Directive on Landfill of Waste (Directive 99/31/EC) demands a reduction of the amount of landfilled organic material. Furthermore, the European Union (EU) end-of-life-vehicle (ELV) directive requires that 85%, by weight, of the materials used in each car and light truck built for the 2015 model year and beyond must be reusable or recyclable. In the future, composites that are fully recyclable are thus needed. The Connora recycling process, using mild conditions, represents a very important innovation and alternative for the end-of-life of thermoset composites.

The LCA method is a very useful tool for companies, because it helps to find out the weakness of their processes and the possible innovations that need to be undertaken. In the case of the Connora process, it was evident that recycling and reusing the carbon fibres are the most important aspects in terms of environmental and economic savings. It is also very important to examine the conditions of the recycled fibres, i.e., if extra treatments are required, or if they are “ready to use”. The mild conditions operated by the Connora process guarantees very little chemical damage on the fibres. However, another aspect is to be taken into account, and it is related to the mechanical mixing of the composite during the process, which could affect the macroscopic shape, especially for woven fibres. Connora technologies are already studying a new system to overcome this technical aspect and guarantee the good quality of the recovered fibres that will be collected on a “ready to use” level.

## Figures and Tables

**Table 1:** Materials consumption for each phase of the recycling process.

Materials (Kg)			
Phase	Water	Acetic Acid	NaOH
*Dissolving*	54.44	18.15	0.00
*PPT*	68.27	0.00	11.98
*Washing*	255.60	0.00	0.00
*Drying*	0.00	0.00	0.00
Total	378.31	18.15	11.98

**Table 2:** Electricity consumption calculation for each phase of the recycling process.

Equipment and Phase		Power (W)	Time (h)	Energy (KJ)
**Dissolving**			3	27,296.6
**Thermoplastic recovery**				
*PPT*	Mixer	negligible		negligible
*Washing*	Centrifuge	2238	1.97	15,889.8
*Drying*	Vacuum	746	35.5	95,338.8
	Tumbler	1119	35.5	143,008.2
	Heater	800	35.5	102,240.0
**Total energy consumption**				
				383,773.4

**Table 3:** Representative properties of the reclaimed epoxy-thermoplastic from recyclable epoxy resins.

Glass transition temperature (*T*_g_)	40–60 °C
Melting temperature (*T*_m_)	120–140 °C
Tensile modulus	2.4 GPa
Tensile strength	57 MPa
Elongation	45%
Shore D hardness	80

**Table 4:** Inventory analysis of the Connora recycling process.

**Input**	**Quantity**
*Dissolution*	
Acetic acid	18 kg
NaOH	12 kg
Water	55 kg
*Thermoplastic precipitation (PPT) and washing*	
Water (PPT)	70 kg
Water (washing)	225 kg
*Total electricity consumption*	383,773 kJ
**Output**	
CF recovered	22.49 kg
CF waste	0.23
Thermoplastic recovered	12.33 kg
Thermoplastic waste	0.45
Waste water production	380 kg

**Table 5:** Inventory analysis of the composite production using the VRTM technique.

Input	Quantity
Composite	35.5 kg
Materials	18 kg
Matrix (epoxy + Recyclamine^®^)	12.78 kg
Carbon fibres	22.72 kg
Process (VRTM)	
Electricity for vacuum pump	4.46 kWh
Electricity for oven	7.14 kWh
*Consumables and scrap:*	
Vacuum bag (Nylon 6,6)	1.228 kg
Breather (PET)	2.579 kg
Tacky tape (synthetic rubber)	19.11 kg
Aspiration tubes (PA 6,6)	1.256 kg
Total electricity consumption	383,773 kJ
Transport	
Matrix	10,500 km (USA)
Carbon fibres	2224 km (Germany)
Vacuum bag (Nylon 6,6)	1354 km (Milan, Italy)
Breather (PET)	1354 km (Milan, Italy)
Tacky tape (synthetic rubber)	2224 km (Germany)
Aspiration tubes (PA 6,6)	1271 km (Varese, Italy)

**Table 6:** Cumulative Energy Demand.

Recovered Item	Amount	Cumulative Energy Demand (CED)
Carbon fibres	22.49 kg	14.8 GJ
PAN	22.49 kg	2.8 GJ

**Table 7:** Avoided impact of recycling 35.5 kg of CF composite through the Connora process: comparison of recovering the carbon fibres as PAN or as reusable CF. Method CML 2000.

Impact Category	Unit	Pan	CF
Abiotic depletion	Kg Sb eq	−0.8	−6.3
Acidification	Kg SO_2_ eq	0.1	−1.7
Eutrophication	Kg PO_4_^3−^ eq	0.1	−0.6
Global warming (GWP100)	Kg CO_2_ eq	−84.5	−779.0
Ozone layer depletion (ODP)	Kg CFC-11 eq	0.0	0.0
Human toxicity	Kg 1,4-DB eq	−49.4	−89.7
Fresh water aquatic ecotoxicity	Kg 1,4-DB eq	−36.6	−82.5
Marine aquatic ecotoxicity	Kg 1,4-DB eq	−82,524.2	−190,801.6
Terrestrial ecotoxicity	Kg 1,4-DB eq	0.0	−0.7
Photochemical oxidation	Kg C_2_H_4_ eq	0.0	−0.1
